# Spatial–temporal pattern and risk factor analysis of bacillary dysentery in the Beijing–Tianjin–Tangshan urban region of China

**DOI:** 10.1186/1471-2458-14-998

**Published:** 2014-09-25

**Authors:** Gexin Xiao, Chengdong Xu, Jinfeng Wang, Dongyang Yang, Li Wang

**Affiliations:** China National Center for Food Safety Risk Assessment, Beijing, 100022 China; State Key Laboratory of Resources and Environmental Information System, Institute of Geographic Science and Natural Resource Research, Chinese Academy of Sciences, Beijing, 100101 China; Key Laboratory of Surveillance and Early Warning on Infectious Disease, Chinese Center for Disease Control and Prevention, Beijing, 102206 China; College of Environment and Planning, Henan University, Kaifeng, 475004 China

**Keywords:** Bacillary dysentery, Epidemiologic feature, Space-time risk analysis, Risk factors

## Abstract

**Background:**

Bacillary dysentery remains a major public health concern in China. The Beijing–Tianjin–Tangshan urban region is one of the most heavily infected areas in the country. This study aimed to analyze epidemiological features of bacillary dysentery, detect spatial-temporal clusters of the disease, and analyze risk factors that may affect bacillary dysentery incidence in the region.

**Methods:**

Bacillary dysentery case data from January 2011 to December 2011 in Beijing–Tianjin–Tangshan were used in this study. The epidemiological features of cases were characterized, then scan statistics were performed to detect spatial temporal clusters of bacillary dysentery. A spatial panel model was used to identify potential risk factors.

**Results:**

There were a total of 28,765 cases of bacillary dysentery in 2011. The results of the analysis indicated that compared with other age groups, the highest incidence (473.75/10^5^) occurred in individuals <5 years of age. The incidence in males (530.57/10^5^) was higher compared with females (409.06/10^5^). On a temporal basis, incidence increased rapidly starting in April. Peak incidence occurred in August (571.10/10^5^). Analysis of the spatial distribution model revealed that factors such as population density, temperature, precipitation, and sunshine hours were positively associated with incidence rate. Per capita gross domestic product was negatively associated with disease incidence.

**Conclusions:**

Meteorological and socio-economic factors have affected the transmission of bacillary dysentery in the urban Beijing–Tianjin–Tangshan region of China. The success of bacillary dysentery prevention and control department strategies would benefit from giving more consideration to climate variations and local socio-economic conditions.

## Background

Bacillary dysentery remains a major public health concern in China [1]. The disease is a severe form of shigellosis and is caused by infection with Shigella bacteria [2]. The bacteria are primarily transmitted via the fecal-oral route [3]. The major symptoms of bacillary dysentery are acute diarrheal episodes, with at least one of the following: fever, abdominal pain, tenesmus, tenderness in the left lower quadrant, and bloody or mucus stool [4]. Worldwide, there are 165 million cases of bacillary dysentery, and 1.1 million cases of death caused by bacillary dysentery every year [5]. In China, approximately 269,299 bacillary dysentery cases were reported in 2009, with an incidence rate of 20.28 per 100,000 [6, 7]. Bacillary dysentery is the third leading notifiable disease in China, following tuberculosis and hepatitis B [6].

The results of many studies have indicated that climate variations have an important part in transmission of the disease, and more research has recently been focused on this issue [7, 8]. The replication and survival of the pathogens in the environment are directly affected by temperature [9]. Precipitation can contaminate drinking water, especially in rural areas with poor water supplies and sanitation infrastructure [10]. Weather conditions can also affect daily lifestyle habits. For example, individuals are less likely to go outdoors during windy environmental conditions. As well as meteorological factors, socio-economic factors are relevant to the epidemiology of bacillary dysentery. For example, the transmission of the disease increases in overcrowded environments with poor sanitation [10]. Several studies have examined the effects of climate on bacillary dysentery. However, to our knowledge no studies have been published that examine the effect of climate in combination with socio-economic factors in China.

The Beijing–Tianjin–Tangshan urban region is one of the three major urban agglomerations in China, and encompasses an area of 43,107.54 km^2^. It has a population of 41.87 million, located in a temperate monsoon climate zone with high climatic variation. In recent years, the incidence of bacillary dysentery has been significant higher in this region compared with other areas. An exploration of the spatial-temporal pattern and factors that affect the incidence of bacillary dysentery would aid in the identification of high-risk areas, and thus guide appropriate allocation of public health resources for better disease control and prevention.

## Methods

### Materials

**Figure 1:**
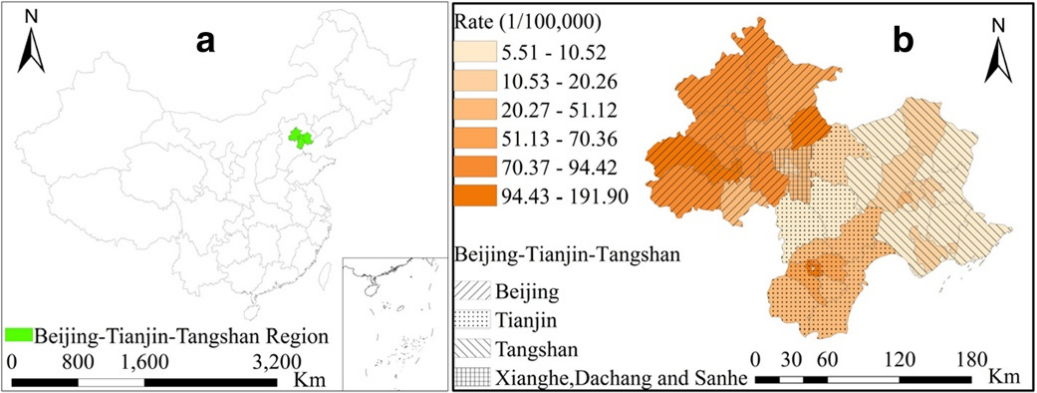
**Geographic location of the Beijing–Tianjin–Tangshan urban region in China (a), study region and disease rate of bacillary dysentery (b).**

**Figure 2:**
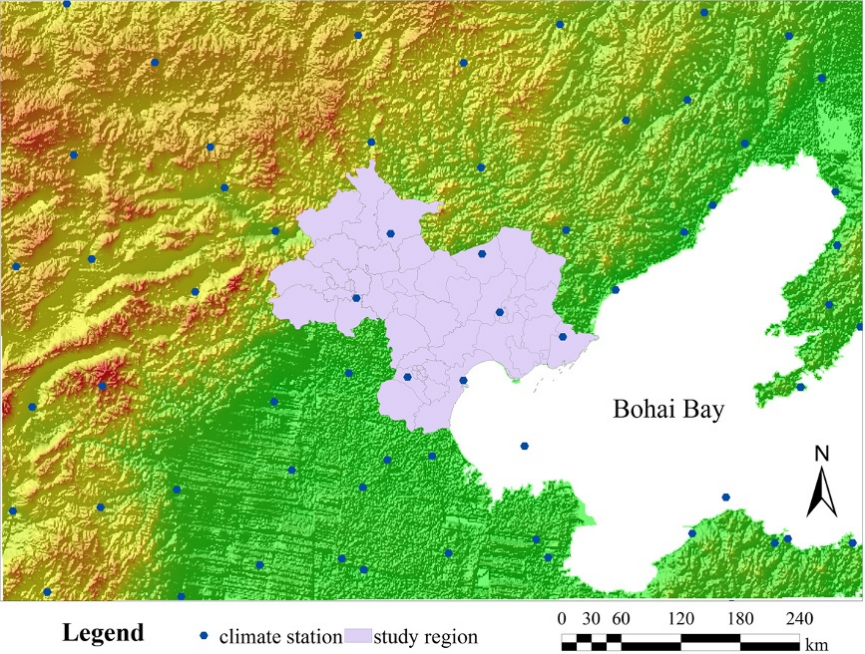
**Distribution of meteorological stations and terrain around the study region.**

Beijing–Tianjin–Tangshan mainly includes Beijing, Tianjin, and Tangshan cities, which include 49 counties (Figure 1). Most of the region included in the study is located on the plain of North China. This region is part of the sub-humid monsoon climate zone, with a mean annual precipitation between 550 and 750 mm. Distinctive temperature and precipitation differences between seasons result from the monsoon climate. Northwest winds from high latitudes result in cold and dry winter conditions. Southeast winds from the pacific result in warm and moist conditions during the summer season.January 2011 to December 2011 statistics on monthly bacillary dysentery cases in Beijing–Tianjin–Tangshan were used for the study. These data were sourced from the daily reported cases from the Chinese Center for Disease Control and Prevention. The total number of cases in the dataset of the reporting system was 28,765, and the valid record number used in the calculation of the study was 28,682. There was a difference of 0.2%, which is a result of missing information in the address field in a small number of records. Data on risk factors relating to socio-economic variables were also collected. These data included population density and gross domestic product (GDP) per capita for each county in the study area, which were obtained from the 2011 Statistical Yearbook. Monthly meteorological data from January 2011 to December 2011 were obtained from the China Meteorological Data Sharing Service System. These data included monthly average air temperature, monthly precipitation, and monthly sunshine hours. The spatial distribution of meteorological stations around the study region was presented in Figure 2. The monthly station value was interpolated to the centroid of each county using the Inverse Distance Weighting (IDW) method.

### The scan statistics

Two scan statistics that provided complementary information were used for the analysis. The purely spatial scan statistic was used to determine the geographical area with the highest risk. The space-time permutation scan statistic was used to find space-time outbreaks that are adjusted for, and are therefore not the result of purely temporal or purely spatial variation.

### (a) Purely spatial scan statistic

SaTScan spatial scan was used to determine the geographical area of bacillary dysentery with the highest risk [11, 12]. For this method, many overlapping circles were used to define the scanning window, and each window was a possible candidate for a spatial cluster. Each circle represented the geographical area of the potential cluster. The area of the circle varied from zero to the maximum specified cluster size of the total risk population. The log likelihood ratio (*LLR*) statistic was calculated for each window. The scan statistic was the maximum likelihood ratio over all the windows, which corresponded to the most likely cluster. Because the number of cases was assumed to follow a Poisson model, the likelihood ratio (*LLR)* for a window was expressed as:


where *n*_*z*_ was the number of cases inside a window, *n*_*G*_ was the total number of cases, and *μ*_*z*_ was the expected number of cases inside the window. The *LLR* value was ranked in decreasing order and the largest *LLR* value was defined as the most likely cluster. The *p*-value for the scan statistic was calculated using Monte Carlo hypothesis testing.

### (b) Space-time permutation scan statistic

The SaTScan space-time permutation scan statistic was used to detect outbreaks of bacillary dysentery in counties over time [13]. The results of this method were depicted as a cylinder. The circle at the bottom of the cylinder represented the geographical area, and the height of the cylinder represented the time period. The spatial–temporal scan was used to test all possible circles for the bottom of the cylinder and all possible time periods for the height, each being a possible candidate for an outbreak. The area of the bottom circle was varied from zero to the maximum specified cluster size of the total cases. The height of the cylinder ranged from a single time period to a defined maximum number of time periods. The statistic Poisson generalized likelihood ratio (*GLR*) was calculated for each window. Among all of the cylinders evaluated, the one with the maximum *GLR* was the space–time cluster of cases that was the primary candidate for a true outbreak. The value of *GLR* was expressed as:


where *c*_*A*_ and *μ*_*A*_ were the observed and expected number of cases in the cylinder, respectively. *C* was the total number of observed cases. A *p*-value for the scan statistic was calculated using Monte Carlo hypothesis testing.

### Spatial panel model

Compared with traditional models using only cross-sectional data, data used in spatial panel models are generally more informative. They contain more variation and less collinearity among the variables. These models result in a greater availability of degrees of freedom, which increases efficiency in the estimation [14]. Spatial data also usually show some autocorrelation [15–17], because spatial panel models consider interaction between spatial units. The spatial lag model is one of the commonly used spatial panel models. It assumes the dependent variable depends on the values of the variables in adjacent spatial units. The spatial panel model was expressed as:


where *i* and *t* were index spatial and time dimensions, respectively; *y*_*it*_ was the dependent variable at spatial unit, *i*, and time, *t*; *x*_*it*_ was the observation for the independent variable at *i* and *t*; *β* was the spatial regression coefficient explaining the relationship between the independent and dependent variables; *ϵ*_*it*_ was the error term with zero mean and equal variance and was assumed to have a normal distribution; and *μ*_*i*_ represented the spatial specific effects in different spatial units. *ρ* was the spatial autoregressive coefficient reflecting the spatial neighborhood effects. For *ρ∈* [0,1], a high value indicated strong spatial autocorrelation, and a low value indicated weak spatial autocorrelation. If *ρ = 0,* then the spatial panel model degenerated to a traditional pane model. *w*_*ij*_ was a spatial weights matrix, which indicated the spatial neighborhood relationship between regions in the dataset [14]. For example, in the order of one neighborhood matrix, if region *i* was directly adjacent to region *j*, *w*_*ij*_ = 1, and if not, *w*_*ij*_ = 0.

## Results

### Epidemiologic characteristics

**Figure 3:**
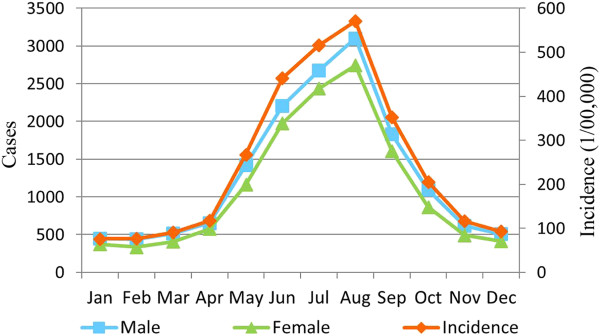
**Monthly incidence and number of cases for males, females, and total population.**

During the winter season (December, January, February), there were approximately 500 cases of bacillary dysentery in both males and females (Figure 3). The corresponding incidence of the disease was close to 100/10^5^ (Figure 3). The incidence began to increase rapidly in April and reached a peak value of 571.10/10^5^ in August (3101 cases in males and 2,742 cases in females). Throughout the entire year, the number of cases in males was slightly higher compared with number of cases in females .

**Figure 4:**
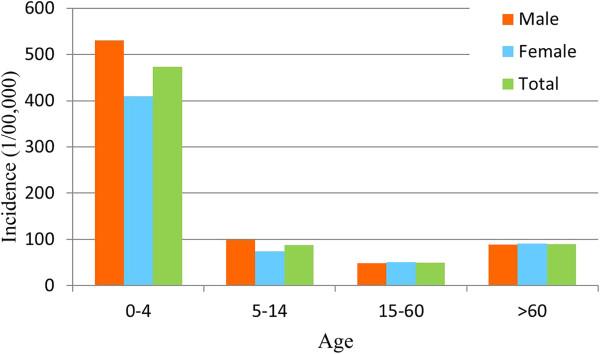
**Disease rates for different ages and genders.**

**Figure 5:**
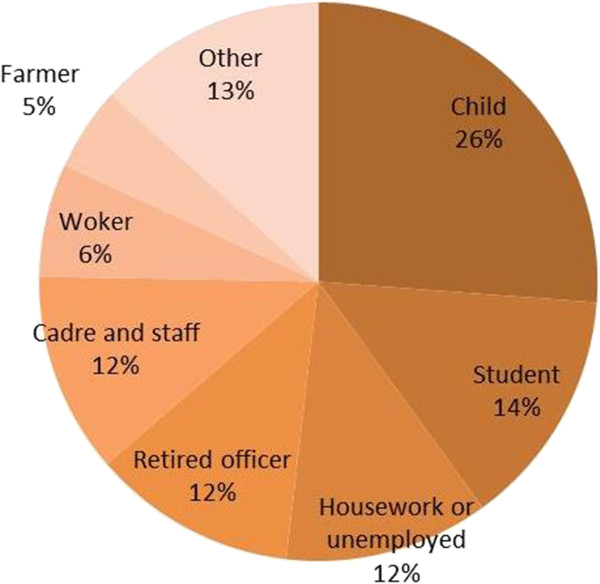
**Cases of disease in different occupations.**

The incidence differed significantly across different age groups. In the age group ‘under 5’ years, the incidence was 473.75/10^5^, which was much higher compared with the other age groups (Figure 4). In this age group, the incidence rate for males was 530.57/10^5^ and the rate for females was 409.06/10^5^. In contrast, the incidence was <100/10^5^ in the other age groups of 5–14, 15–60, and >60 years. For example, the incidence in males was 99.10/10^5^ in the 5–14 year age group, and the incidence for females in this age group was lower, at 74.10/10^5^. However, in the 15–60 years, and >60 years age groups, the incidence in males was slightly lower compare with in females.The cases in children accounted for the largest proportion of cases, and were 26% of all bacillary dysentery cases reported during the study period (Figure 5). For occupational groups, the percentage of cases in the occupational groups of ‘student’ , ‘housework or unemployed’ , ‘retired officer’ , ‘cadre/staff’ , ‘worker’ , ‘farmer and ‘others’ occupational groups were 14%, 12%, 12%, 6%, 5%, and 13%, respectively.

### Spatial clusters

**Figure 6:**
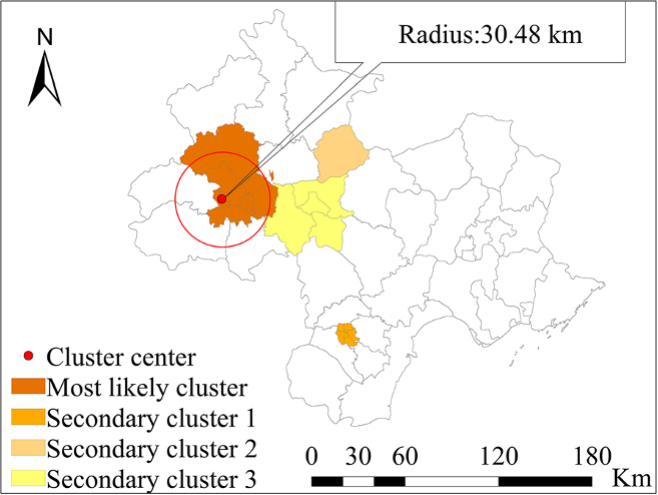
**Spatial clusters of bacillary dysentery in the total population at risk.**

A SaTScan spatial scan was used to detect spatial clusters. The spatial units in the Beijing–Tianjin–Tangshan region included 49 counties. The ‘population at risk’ was set to 30% of the total population at risk to explore transmission characteristics of the disease for different spatial scales within the region (Table 1). The most likely clusters included seven districts. The cluster center was in the Shijingshan district (116.17° E, 39.93° N) of Beijing, with a cluster radius of 30.48 km (Figure 6). The average annual incidence inside the spatial window was 118.5/10^5^ with a relative risk (RR) value of 2.18 (P = 10^-17^).

**Table 1:** **Results for spatial cluster analysis of bacillary dysentery**

Cluster	Center	Number of counties	Observed	Expected	***LLR***	***RR***	***P***value
1*	(116.17° E, 39.93° N)	7	13711	8490.57	2092.42	2.18	0.000
2	(117.19° E, 39.12° N)	6	5325	3471.51	494.54	1.66	0.000
3	(117.14° E, 40.21° N)	1	655	389.70	76.06	1.7	0.000
4	(117.05° E, 39.74° N)	4	1656	1352.54	33.44	1.24	0.000

Note: * indicates the most likely cluster, the others indicate secondary cluster. *LLR* is log likely ratio, and *RR* is relative risk.

### Spatial–temporal outbreak

**Figure 7:**
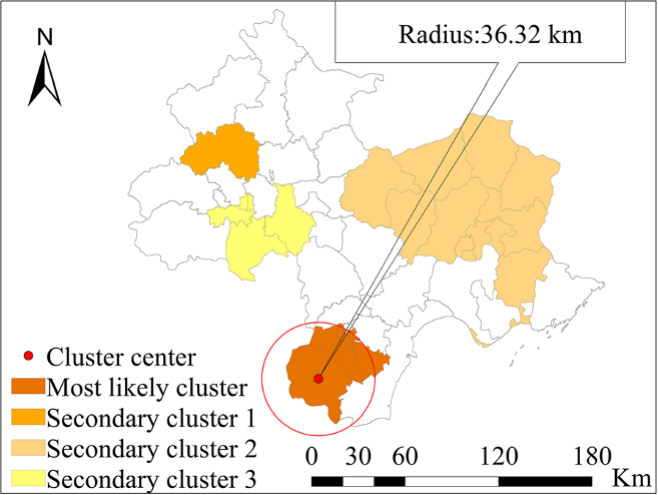
**Locations of detected spatial-temporal outbreaks of bacillary dysentery.**

The space-time permutation scan statistic was used to locate outbreaks of bacillary dysentery in counties over time, and it can adjust the purely temporal or spatial variation in risk. To detect the disease outbreak characteristics in different spatial-time scales within the region, the maximum spatial circle size was set to 30% of the total cases, and a maximum temporal length of 4 months was included in the calculation (Table 2). We used this period to reflect the seasonal effect. The strongest signal was in May 2011, and included eight counties that were mostly located in the suburban areas of Tianjin. The center of the signal was at 116.96° E, 38.86° N. The radius was 36.32 km. This signal consisted of 1,630 observed cases that occurred during a 2-month period when 1,313.02 cases were expected to occur (RR = 3.65). With a very small *p*-value close to 0, this result indicates that a signal of this magnitude was unlikely to be due to random variation. The signal immediately preceded a sharp increase in cases in the entire region from May to August 2011 (Figure 7). Both of the second and fourth signals that followed were centered in suburban Beijing during February to April 2011 and January to February 2011, respectively. The third single was located in Tangshan during July to September 2011. The probable causes of the above outbreaks are discussed below.

**Table 2:** **Results for spatial-temporal outbreak detection of bacillary dysentery**

Signal ID	Center	Start date	End date	Number of counties	Observed	Expected	***RR***	***P***value
1*	(116.96° E, 38.86° N)	2011/5	2011/6	8	1630	1313.02	3.65	0.000
2	(116.21° E, 40.22° N)	2011/2	2011/4	1	211	137.45	2.79	0.000
3	(118.36° E, 40.23° N)	2011/7	2011/9	12	618	490.99	1.4	0.000
4	(116.41° E, 39.65° N)	2011/1	2011/2	5	505	407.83	1.9	0.001

Note: *indicates the most likely cluster, the others indicate secondary cluster, and *RR* is relative risk.

### Risk factors

Disease incidence was a dependent variable in a spatial panel model. Four independent variables were examined for the January 2011 to December 2011 period. They including four meteorological factors (i.e., monthly average temperature, monthly average relative humidity, monthly cumulative rainfall, and monthly total sunshine). The splm package in the R 3.02 statistical software program was used for the spatial panel modeling [18].

The results of the spatial panel model are presented in Table 3. The coefficient of spatial dependence was 0.593, which was highly significant in the models (*p-*value < 0.001) and indicated the presence of neighborhood effects. The risk factor analysis indicated that there was a positive association between bacillary dysentery and temperature and precipitation (*p-*values < 0.05). These results indicated that by controlling the spatial effect, a 1°C rise in average temperature was related to a 10.6% (95% CI: 9.1% – 12.1%) increase in the number of cases of bacillary dysentery. A 1 mm increase in precipitation was related to a 0.5% (95% CI: 0.3% – 7%) increase in the number of cases of bacillary dysentery. There was a negative association between bacillary dysentery and sunshine hours, but this relationship was not statically significant (*p-*value > 0.05).

**Table 3:** **Results of spatial panel model using meteorological risk factors**

Variables	Coefficient	S.E	***t***	***p***
Spatial weight	0.593	0.0406	14.580	0.000
Average temperature (°C)	0.106	0.015	6.92	0.000
Precipitation (mm)	0.005	0.002	2.132	0.033
Sunshine hours (hour)	-0.005	0.003	-1.74	0.082

Note: S.E. is standard error of estimated coefficient.

Socio-economic risk factors also contributed to the spatial temporal distribution of the disease. However it was difficult to obtain data on the monthly variation in these factors, so spatial panel modeling could not be used for the analysis. A spatial lag model (SLM) was used for analysis of these factors. Disease incidence was a dependent variable and the two socio-economic factors (including population density and GDP per capita) were independent variables. The SLM was implemented using GeoDa software (GeoDa Center, Tempe, AZ, USA).

The results of the SLM are presented in Table 4. The coefficient of spatial dependence was 0.52, which was highly significant (*p*-value < 0.001), and indicated the presence of neighborhood effects. The risk factor, population density, was highly significant (*p-*value < 0.05). By controlling the spatial effect, population density was positively associated with the disease. The disease incidence increased by 240% (95% CI: 180–300%) with a population density increase of 1000 persons per square kilometers. GDP per capita was negatively associated with the disease incidence. Disease incidence decreased by 20% (95% CI: 10–30%) with a per capita GDP increase of 1 million yuan.

**Table 4:** **Results of spatial lag model using socio-economic risk factors**

Variables	Coefficient	S.E	***p***
spatial weight	0.52	0.14	0.000
GDP per capita(1000 Yuan)	-0.20	0.10	0.066
Population density (1000 person/km^2^)	2.40	0.60	0.000

Note: S.E. is standard error of estimated coefficient.

## Discussion

Bacillary dysentery remains a major public health concern in China. The Beijing–Tianjin–Tangshan region is the largest urban agglomeration in north China, In recent years, it has experienced a notably high incidence of bacillary dysentery compared with other areas. This study explored the epidemiologic characteristics of the disease, and detected the high-risk areas using scan statistics. The associations between bacillary dysentery and meteorological variables, as well as socio-economic factors, were examined. The results indicated that (1) the incidence of bacillary dysentery in the region was still high, especially in children; (2) the risk area was mainly located at the areas with high population densities and disease outbreaks were mainly distributed in suburban district areas during festivals and holidays; (3) meteorological factors have significantly affected the transmission of dysentery. Population density has also had a significant influence.

The exploration of the epidemiologic characteristics revealed that the incidence of bacterial dysentery changed with the seasons during the 1-year study period. It was higher during summer and fall, with the peak appearing in August. The incidence in children was much higher compared with other age groups. These findings are similar to the findings of previous studies conducted in other regions of China [4]. The seasonal variation in incidence may be associated with meteorological risk factors, which will be discussed below. The difference in incidence between males and females <5 years of age may be because boys are more active than girls, and thus would have more opportunities to be exposed to environments containing bacteria.

The transmission of Shigella can be affected by many factors (e.g., local weather conditions, socio-economic conditions, dietary habits, personal hygiene, and susceptibility to different pathogen strains). Climate variations are considered to be one of the key environmental factors affecting the incubation and survival of Shigella. We found that a 1°C increase in average temperature was associated with a 10.6% increase in bacillary dysentery incidence. This result was consistent with the results of previous studies. Zhang et.al. found that in Jinan City in northern China, bacillary dysentery incidence increases by 12% with a 1°C increase in maximum or minimum temperature [10]. Checkley et al. found that diarrhea incidence increased by 8% per 1°C increase in mean ambient temperature [19]. Bacteria replicate faster in a higher temperature environment, and food is prone to deteriorate in these environments.

We found that precipitation is another climate factor that affects the transmission of bacillary dysentery. A 1 mm increase in precipitation was associated with a 0.5% increase in bacillary dysentery incidence. Precipitation may exacerbate the transmission of enterovirus-related diseases by affecting replacement of pathogens in contaminated drinking water. However, the results of some studies have drawn opposite conclusions regarding the effect of precipitation on bacillary dysentery. Huang et al. found that there was a positive association between precipitation and the spread of bacillary dysentery [20]. Li et al. found that there was a negative relationship [7].

Socio-economic conditions are another group of factors that affect the transmission of the disease. In this study, GDP per capita was selected as a proxy variable for hygiene behavior or public health condition of a region. Population density was selected as a proxy variable for frequency of contact between people, which will accelerate the transmission of viruses. The result indicated that GDP per capita has a negative association with the disease. The disease incidence decreased by 20% with a GDP per capita increase of 1 million yuan. Population density was positively associated with disease incidence; the incidence decreased by 240% with a population density increase of 1000 persons per square kilometers. This result suggests that improvement in living conditions would reduce disease transmission. Preventative strategies should be concentrated in areas with high population densities.

The results of the SaTScan indicated that the most likely spatial clusters were mainly located in the urban regions of Beijing (RR = 2.18, *p-*value = 10^-17^). The population density was very high in this area. The result was consistent with the finding of the factor analysis using SLM, in which the population density was positively associated with bacillary dysentery. The outbreak detection analysis revealed that the main outbreaks located in suburban Beijing and Tianjin and some areas of Tangshan during April and May 2011 coincided with the two important Chinese festivals and holidays (i.e., the Spring Festival and the May Day holiday). Migrant workers mainly reside in the suburban areas, where the socio-economic conditions are relatively poor compared with conditions in the urban areas. Thus, when combined with the peak tourist season, conditions are created that are favorable for a disease outbreak. The effectiveness of public health department interventions and prevention strategies would benefit from clearly defining risk areas and space-time locations for outbreaks.

There were also some limitations of this study. There have been improvements in the reporting of notifiable infectious diseases by Chinese medical facilities, but under-reporting may have occurred during the disease surveillance process [4]. For example, although the notifiable infectious disease surveillance system included the mobile population, some individuals (e.g., tourists who visited for only a short period of time) may be hospitalized after they return home. The proportion of the mobile population in the total reported bacillary dysentery cases could not be assessed using the dataset. Underreporting would weaken the associations between risk factors and bacillary dysentery incidence. Therefore, it is possible that the results of this study represent an underestimate of the true values. In future studies, the under-reporting rate should be estimated and included in the study. A second study limitation was that bacillary dysentery may be significantly affected by micro-environments. A county-level spatial scale was used in this study because no data were available for smaller areas (e.g., the village or town level). The spatial scale used may have obscured some factors via the ecological fallacy effect [21].

## Conclusion

In summary, bacillary dysentery was widespread throughout the Beijing–Tianjin–Tangshan region during 2011, and represents a serious threat to human health in this region. Effective public health measures can be implemented if they are based on a deep understanding of the epidemic characteristics, spatial-temporal clusters, and factors that affect bacillary dysentery incidence. We found that meteorological and socio-economic factors have affected the transmission of bacillary dysentery. The success of bacillary dysentery prevention and control department strategies would benefit from giving more consideration to climate variations and local socio-economic conditions.
